# AHAT-Fin: an adaptive hybrid attention and market-state-aware framework for financial time-series forecasting

**DOI:** 10.3389/frai.2026.1975632

**Published:** 2026-10-09

**Authors:** Yingli Gong, Dongdong Lv, Zhijian Duan

**Affiliations:** 1 School of Supply Chain Management, Ningbo Polytechnic University, Ningbo, Zhejiang, China; 2 School of Artificial Intelligence, Ningbo University of Technology, Ningbo, Zhejiang, China

**Keywords:** adaptive hybrid attention, financial time series forecasting, iTransformer, market state perception, quantitative investment

## Abstract

Accurate financial time-series forecasting is essential for quantitative investment. However, existing methods often perform inadequately when handling the complex dynamics of financial data, including non-stationarity, volatility clustering, and market regime transitions. These characteristics can adversely affect both forecasting accuracy and the quality of investment decisions. To address these challenges, this study develops AHAT-Fin, an improved Transformer-based forecasting framework that integrates adaptive hybrid attention with market state perception. AHAT-Fin integrates dual-path adaptive hybrid attention, generalized autoregressive conditional heteroskedasticity (GARCH)-based dynamic volatility adjustment, and hierarchical market-state perception based on multi-window long short-term memory (LSTM) networks. The model is evaluated using daily trading data from seven representative U.S. stocks and selected constituents of the CSI 300 Index. The results show that AHAT-Fin generally outperforms the benchmark models, achieving an average reduction of 20.2% in mean squared error, an 8.4% improvement in directional accuracy, and a 63.4% increase in the Sharpe ratio. Module-level evaluation further confirms that all three components contribute to the overall predictive performance, with the market state perception module playing a particularly important role in improving risk-adjusted investment performance. Visual analyses of attention weights and market state identification further demonstrate the model’s ability to extract multidimensional financial features and capture transitions across market states. These findings demonstrate the potential of combining volatility-aware modeling, adaptive attention, and market-state perception for financial forecasting and quantitative strategy design.

## 1 Introduction

Financial markets are complex adaptive systems in which price formation is jointly influenced by macroeconomic conditions, market liquidity, investor behavior, and unexpected events. As a result, financial time series often exhibit pronounced non-stationarity, volatility clustering, and regime-switching characteristics. Engle introduced the autoregressive conditional heteroskedastic-ity (ARCH) model to capture the time-varying nature of conditional variance in financial time series (Engle, 1982), while Bollerslev (1986) subsequently developed the GARCH model by incorporating both past shocks and lagged conditional variances into volatility modeling, thereby establishing an important foundation for financial risk analysis. Financial markets are not governed by a single, stable data-generating process; their dynamic characteristics may change over time. Hamilton’s Markov regime-switching model provided an important theoretical framework for describing such structural changes (Hamilton, 1989). When market conditions shift substantially or extreme events occur, the distribution of asset returns, volatility levels, and dependence structures among financial variables may change simultaneously. Such changes pose considerable challenges to forecasting models based on relatively stable data-generating assumptions. Volatility is central to asset allocation, risk assessment, and the estimation of risk measures such as Value-at-Risk (VaR) and Expected Shortfall (ES); accordingly, forecasting accuracy directly affects the ability of financial institutions and investors to identify potential losses and allocate risk capital (Buczynski and Chlebus, 2024; Petrozziello et al., 2022; Wang et al., 2024). Against this background, jointly modeling nonlinear dependencies among financial variables, time-varying volatility, and market regime transitions under complex market conditions has become an important research problem at the intersection of artificial intelligence and financial risk management.

Research on financial time series forecasting and risk characterization has progressively evolved from traditional econometric models toward deep learning and attention-based architectures. Models such as autoregressive integrated moving average (ARIMA), ARCH, and GARCH are able to capture serial dependence and volatility clustering from a statistical perspective. GARCH-type models, in particular, are well suited to describing conditional heteroskedasticity and volatility persistence in financial asset returns (Bollerslev, 1986; Engle, 1982), but they have limitations in modeling complex nonlinear dependencies among high-dimensional financial variables and structural changes. With the development of deep learning, LSTM networks have enhanced the ability of recurrent neural networks to model long-term dependencies through memory cells and gating mechanisms, and have gradually been applied to financial time series forecasting and volatility analysis. Petrozziello et al. (2022) used deep LSTM networks for multivariate volatility forecasting and showed that deep learning models can effectively capture the volatility characteristics of complex financial assets. Sahiner et al. further applied artificial neural networks to market volatility forecasting and evaluated their risk management performance using VaR, ES, and associated backtesting procedures (Sahiner et al., 2023). Taken together, these studies indicate that combining financial volatility structures with deep neural networks may improve the modeling of complex financial risks. However, existing approaches often examine volatility forecasting, tail-risk estimation, and deep network design separately, resulting in a fragmented research landscape.

In recent years, attention mechanisms and Transformer architectures have expanded the methodological foundations for multivariate and long-horizon financial forecasting. The Temporal Fusion Transformer integrates recurrent structures, variable-selection mechanisms, and attention to capture information across multiple time scales and support multi-step forecasting (Lim et al., 2021).

In financial applications, hybrid LSTM–Transformer models combine local temporal patterns with long-range dependencies, thereby improving the representation of complex financial dynamics (Kabir et al., 2025). Recent studies have also explored point and interval forecasting to characterize predictive uncertainty, as well as attention refinement and transfer learning to improve forecasting across financial instruments (Qi et al., 2025; Feng and Sinchai, 2025). More recently, dual-attention structures have been introduced to jointly model multidimensional relationships in financial time series, highlighting the potential of multi-channel attention for complex financial forecasting (Hadizadeh et al., 2025). Transformer-based methods have also moved beyond price prediction toward volatility forecasting and financial risk assessment. For example, Mishra et al. combined Transformer and Multi-Transformer architectures with GARCH and LSTM models, demonstrating the ability of attention-based architectures to represent complex market dynamics (Mishra et al., 2024). These studies provide an important methodological foundation for applying deep attention-based models to financial risk forecasting.

In addition to developing new forecasting architectures, researchers have increasingly focused on integrating structural knowledge from financial econometric models with deep learning. Buczynski and Chlebus (2024) proposed GARCH Net, which combines the conditional variance structure of GARCH with neural networks for Value-at-Risk forecasting of financial assets, enhancing nonlinear modeling capability while preserving the interpretability of conventional volatility models.

Wang et al. (2024) combined deep quantile regression, generative models, and the heterogeneous market hypothesis to jointly forecast VaR and ES, providing further evidence of the potential of deep learning to characterize tail risk in asset-return distributions. Léber and Egyed (2026) further incorporated market sentiment information into a hybrid GARCH-LSTM framework for stock volatility and VaR forecasting. Taken together, these studies suggest that combining financial volatility structures with deep neural networks can improve the identification and modeling of complex financial risks. However, existing studies often examine volatility forecasting, tail-risk estimation, and deep network design separately, resulting in a relatively fragmented research landscape.

Building on the literature reviewed above, three issues remain to be addressed in AI-based financial forecasting. First, existing models often emphasize either temporal dependencies or inter-variable dependencies. Some Transformer-based models have improved the modeling of multivariate relationships, relatively few dynamically coordinate the importance of cross-variable and temporal information as market conditions change. Second, financial data exhibit pronounced volatility clustering and conditional heteroskedasticity, while many deep learning models rely on normalization procedures that do not explicitly account for time-varying volatility, including conventional Z-score normalization and layer normalization. The distribution of the model inputs may change, potentially undermining forecasting stability during periods of elevated volatility. Third, although recent studies on volatility, VaR, and ES forecasting have begun to employ deep learning to improve risk measurement performance (Léber and Egyed, 2026; Wang et al., 2024), most models do not explicitly incorporate market regime transitions into a unified forecasting framework. As a result, they may not fully capture the simultaneous changes in variable dependencies, the relevance of historical information, and risk distributions as markets move across oscillating, trending, and abnormal high-volatility market regimes.

Accordingly, the central research gap addressed in this study concerns how to construct an AI-based forecasting framework that jointly accounts for cross-variable dependencies, time-varying volatility, and market-regime changes, rather than merely reducing forecasting errors. These three characteristics are closely interrelated. Changes in market volatility may coincide with a reconfiguration of dependencies among financial variables, while regime transitions may alter the relative importance of different historical periods and variables in explaining future market movements. If a model relies on a fixed information extraction structure, relationships learned from normal market conditions may not remain effective for forecasting during periods of elevated volatility or structural breaks. Existing deep learning models for financial risk have demonstrated the potential of artificial intelligence for volatility and tail-risk forecasting, but further research is needed to integrate variable dependencies, conditional volatility, and market regime transitions within a unified adaptive learning framework.

To address these issues, this study proposes AHAT-Fin, a financial forecasting framework built upon the iTransformer architecture and integrating adaptive hybrid attention with market-state perception. The framework consists of three interrelated core modules. First, a dual-path adaptive hybrid attention mechanism is designed to learn dynamic interactions among financial variables and temporal dependencies along the variable and temporal dimensions, respectively. An adaptive fusion mechanism then adjusts the relative contribution of these two information sources in response to changing input patterns. Previous studies on multi-scale attention, hybrid Transformers, and dual-attention structures suggest that jointly modeling different information dimensions can enhance feature representation in complex time series (Feng and Sinchai, 2025; Hadizadeh et al., 2025). Second, a volatility-aware normalization module inspired by the conditional heteroskedasticity structure of GARCH is developed to incorporate dynamic volatility information into the neural network’s feature-normalization process. In contrast to studies that use GARCH primarily as a standalone model for volatility or VaR forecasting (Léber and Egyed, 2026; Wang et al., 2024), the proposed approach uses conditional volatility information to dynamically rescale the input features, thereby helping the model accommodate volatility clustering and distributional changes during periods of elevated volatility. Third, a multi-scale market state perception module is introduced to extract short, medium, and long-term market features from different time windows and to incorporate regime-transition information, enabling the forecasting network to adjust its behavior across different market conditions.

The main contributions of this study are fourfold. First, a dual-path adaptive hybrid attention mechanism is proposed to integrate variable-wise and temporal attention within a unified architecture. Dynamic weighting is used to regulate the contribution of the two information channels, thereby improving the model’s ability to capture the joint evolution of inter-variable linkages and temporal dependencies. Second, GARCH-based conditional volatility information is incorporated into the deep-learning feature-processing pipeline through a volatility-aware normalization mechanism. This allows the normalization procedure to adjust dynamically to prevailing market volatility and helps the model account for non-stationarity and volatility clustering. Third, a multi-scale market state perception mechanism is introduced to incorporate market information across different time horizons and regime-transition characteristics into the forecasting process, allowing the model to adapt more effectively to oscillating, trending, and highly volatile market conditions. Fourth, model performance is evaluated not only using statistical forecasting measures such as mean squared error (MSE), mean absolute error (MAE), and directional accuracy, but also from the perspective of risk-adjusted returns. Cross-asset tests, and ablation experiments are further used to identify the practical contribution of each core module, providing a technical framework that combines financial structural knowledge with deep learning for financial forecasting and quantitative investment.

## 2 Materials and methods

### 2.1 Data and study design

To evaluate the applicability of the model across different market environments and types of financial assets, this study constructs the research sample using daily trading data from the Chinese and U. S. equity markets. For the U. S. market, representative stocks with different industry characteristics and volatility profiles are selected, while constituent stocks related to the CSI 300 Index are used for the Chinese market. This design increases sample heterogeneity in terms of market structure, industry characteristics, and volatility patterns. The raw data mainly include five basic trading variables: opening price, highest price, lowest price, closing price, and trading volume. The original sample covers both the Chinese and U. S. capital markets, and stocks with different volatility characteristics are used for model comparison and generalization tests.

Data preprocessing combines logarithmic returns with feature scaling. Specifically, logarithmic differencing is first applied to the original price series to remove trend effects. As shown in Equation (1).


$$r_{t}=\ln(P_{t})-\ln(P_{t-1})\;$$ (1)

where $r_{t}$ denotes the log return at time t, and $P_{t}$ denotes the closing price at time t. The data are then standardized using Z-score normalization to ensure a consistent scale across variables. A Box–Cox transformation is applied to reduce skewness in trading volume (Box and Cox, 1964), while a moving-average filter is used to mitigate abnormal fluctuations and noise, thereby improving the quality of the training data.

A dynamic time-series segmentation scheme is adopted, with the historical observation window set to 96 consecutive calendar days and the forecasting horizon set to 24 calendar days. These parameters are determined with reference to financial market cycles and principles of technical analysis. The 96-day window corresponds approximately to a quarterly cycle and is intended to capture medium-term fluctuations and seasonal patterns, whereas the 24-day forecasting horizon is aligned with the decision cycle of quantitative investment while maintaining timeliness and statistical reliability. A one-day rolling window is used to update the data continuously and optimize the use of historical information. The sample is divided chronologically into training, validation, and test sets at a ratio of 7:2:1, with the most recent observations reserved for final testing to assess the model’s adaptability to market conditions.

### 2.2 Overall architecture of AHAT-Fin

To address the non-stationarity, volatility clustering, multivariate dependencies, and changing market states that characterize financial time series, this study proposes AHAT-Fin, a financial time-series forecasting model built upon the iTransformer architecture (Liu et al., 2024). While retaining the multivariate modeling capability of iTransformer, AHAT-Fin incorporates three components: a dual-path adaptive hybrid attention mechanism, a volatility-aware normalization module, and a multi-window market-state perception module. Together, these components are designed to enhance the representation of complex financial market dynamics.

The model uses multivariate observations from the preceding 96 trading days as input. After embedding, the input is processed by the encoder and three core modules for feature extraction and adaptive adjustment. First, the dual-path adaptive hybrid attention mechanism models inter-variable interactions and temporal dependencies along the variable and temporal dimensions, respectively. Second, the volatility-aware normalization module incorporates GARCH-based conditional volatility information and dynamically rescales features in response to changes in market volatility. Third, the multi-window market-state perception module uses LSTM networks with different window lengths to extract short-, medium-, and long-term market features and identify [range-bound, trending, and high-volatility] market states. The resulting representations are then passed to the forecasting head to generate multi-step forecasts of the target series.

AHAT-Fin extends the baseline iTransformer architecture in three ways. First, the dual-path adaptive hybrid attention mechanism jointly models inter-variable interactions and temporal dynamics. Second, the volatility-aware normalization module incorporates GARCH-based conditional volatility information into the feature-processing pipeline, helping the model accommodate volatility clustering and non-stationarity. Third, the multi-window market-state perception module captures market characteristics and their transitions across different temporal horizons. Together, these components enable AHAT-Fin to model complex financial dynamics more effectively.

#### 2.2.1 Dual-path adaptive hybrid attention mechanism

##### 2.2.1.1 Variable-wise attention

Conventional attention mechanisms may not fully capture the complex dependencies among multidimensional financial variables, which can limit the effective use of cross-variable information. Recent studies have therefore begun to explore dual-attention architectures that jointly model heterogeneous financial variables and temporal dependencies, showing their potential to improve financial time-series forecasting performance (Hadizadeh et al., 2025). Building on this line of research, the proposed variable-wise attention mechanism focuses specifically on dynamic interactions among financial variables by constructing a dynamic covariance matrix to characterize their changing relationships.

The mechanism applies attention along the variable dimension to weight multivariate financial features adaptively. Specifically, the input tensor X, which contains both temporal and feature dimensions, is first projected through affine transformations to generate the query, key, and value matrices. As shown in Equation (2):


$$Q_{v}={\mathrm{XW}}_{Q}^{v},K_{v}={\mathrm{XW}}_{K}^{v},V_{v}={\mathrm{XW}}_{V}^{v}$$ (2)

Where $W_{Q}^{v},W_{K}^{v},W_{V}^{v}\in {\mathbb{R}}^{N\times d_{k}}$ denotes a trainable parameter matrix, from which dynamic weights for each feature dimension are obtained through matrix operations. As shown in Equation (3).


$$\begin{array}{c} A_{v}=\text{softmax}(\frac{Q_{v}K_{v}^{T}}{\sqrt{d_{k}}}) \end{array}$$ (3)

The final variable-wise attention output is given by Equation (4):


$$\begin{array}{c} Z_{v}=A_{v}V_{v} \end{array}$$ (4)

This mechanism dynamically captures changes in the relative importance of multiple financial variables and enhances the model’s feature representation capability.

##### 2.2.1.2 Temporal attention

Financial market data exhibit strong temporal dependence, and signals from different historical periods contribute unequally to future price movements. During trend reversals or extreme market events, more recent observations may become more informative for forecasting. The proposed temporal attention mechanism captures dependencies across time horizons and introduces dynamic weighting to improve the representation of temporal features.

The temporal weighting mechanism assigns adaptive weights along the time axis and identifies the historical observations that contribute most to the forecasting target. After variable-wise attention, the feature representation is transposed to construct the query, key, and value tensors along the temporal dimension. As shown in Equation (5).


$$\begin{array}{c} Q_{t}=Z_{v}^{T}W_{Q}^{t},K_{t}=Z_{v}^{T}W_{K}^{t},V_{t}=Z_{v}^{T}W_{V}^{t} \end{array}$$ (5)

The temporal attention weights are computed as follows. As shown in Equation (6):


$$\begin{array}{c} A_{t}=\text{softmax}(\frac{Q_{t}K_{t}^{T}}{\sqrt{d_{k}}}) \end{array}$$ (6)

The final output is given by Equation (7):


$$\begin{array}{c} Z_{t}={\left(A_{t}V_{t}\right)}^{T} \end{array}$$ (7)

This architecture dynamically adjusts the weights assigned to different time windows according to current market conditions and forecast dynamics, enabling the model to capture temporal patterns in financial data more effectively.

##### 2.2.1.3 Adaptive weight fusion strategy

Dual-dimensional feature extraction can capture complex patterns, but effectively integrating information across dimensions remains challenging. Conventional linear combination or feature concatenation may introduce interference between feature representations and limit their complementary effects. To address this issue, an adaptive parameter-tuning module is introduced, in which a temporal convolutional network generates dynamic weighting coefficients so that the fusion strategy can adjust to changing market conditions. A gated recurrent unit is used to estimate the dynamic weight distribution of spatiotemporal features, while a parameter generator based on a fully connected layer and a Sigmoid activation function performs nonlinear weighted integration. Residual connections are incorporated into the fusion architecture to preserve feature information and improve training stability, helping mitigate gradient attenuation while retaining statistical characteristics. This fusion strategy dynamically adjusts the contribution of different features across market conditions, improving the model’s adaptability to complex financial data and enhancing the effectiveness of dual-path attention in financial time series forecasting.

#### 2.2.2 Volatility-aware normalization module

##### 2.2.2.1 GARCH-inspired integration mechanism

This module integrates GARCH-based volatility information with the deep learning framework to address the limited ability of conventional normalization methods to accommodate volatility clustering in financial markets. ARCH and GARCH models were originally developed to characterize time-varying conditional variance and volatility persistence in economic and financial time series (Bollerslev, 1986; Engle, 1982). Building on this principle, the proposed module constructs a dynamic volatility estimation mechanism and incorporates volatility information into the normalization process of the deep learning model.


$$\begin{array}{c} \sigma_{t}^{2}=\alpha_{0}+\sum_{i=1}^{p}\alpha_{i}\epsilon_{t-i}^{2}+\sum_{j=1}^{q}\beta_{j}\sigma_{t-j}^{2} \end{array}$$ (8)

This formulation (8) describes the generation of the conditional variance in a GARCH(p,q) model, where $\sigma_{t}^{2}$ denotes the conditional variance at time *t*, $\epsilon_{t}$ represents the residual series, and $\alpha_{0}$ $\alpha_{i}$ and $\beta_{j}$ are model parameters. Based on this formulation, a dynamic volatility adaptation mechanism is developed. A rolling-window procedure is used to update historical volatility measures and incorporate them into the adjustment of normalization parameters. The mechanism adapts to different market conditions, enhancing sensitivity to abnormal observations during extreme market movements while maintaining computational stability under normal conditions. It is therefore well suited to the volatility clustering and conditional heteroskedasticity commonly observed in financial data. Recent studies have further demonstrated the value of combining volatility structures with deep neural networks for financial volatility and risk forecasting (Buczynski and Chlebus, 2024; Petrozziello et al., 2022).

##### 2.2.2.2 Volatility-aware normalization algorithm

The proposed algorithm extends the GARCH-based framework by introducing a dynamic data preprocessing procedure. Its core idea is to adaptively adjust historical volatility parameters. The implementation consists of two steps. First, a sliding-window procedure is used to estimate historical volatility parameters. Second, a dynamic weight matrix is generated to construct an adaptive normalization scheme. As shown in Equation (9).


$$\begin{array}{c} {\hat{x}}_{t}=\frac{x_{t}-\mu_{t}^{\mathrm{vol}}}{\sigma_{t}^{\mathrm{vol}}\cdot (1+\lambda \cdot {\mathrm{vol}}_{t})} \end{array}$$ (9)

Where ${\hat{x}}_{t}$ denotes the standardized data sequence, $\mu_{t}^{\mathrm{vol}},\sigma_{t}^{\mathrm{vol}}$ represent the sample mean and standard deviation of the volatility-aware indicator, respectively, ${\mathrm{vol}}_{t}$ denotes the volatility measure at time t, and λ is the response coefficient. The statistical parameters are calculated as follows. As shown in Equation (10):


$$\begin{array}{c} \mu_{t}^{\mathrm{vol}}=\frac{\sum_{i=1}^{w}w_{i}^{\mathrm{vol}}\cdot x_{t-i}}{\sum_{i=1}^{w}w_{i}^{\mathrm{vol}}},w_{i}^{\mathrm{vol}}=\exp(\alpha \cdot {\mathrm{vol}}_{t-i}) \end{array}$$ (10)

Where $w_{i}^{\mathrm{vol}}$ denotes the dynamic weighting factor. The model introduces a dynamic volatility-parameter adjustment mechanism that increases the weights assigned to observations during periods of pronounced market turbulence and reduces them under relatively stable market conditions. This adaptive weighting scheme improves the model’s ability to capture fluctuations in financial asset prices and enables dynamic adjustment of risk measures.

##### 2.2.2.3 Non-stationarity handling strategy

This module adopts a hierarchical processing scheme to mitigate non-stationarity in financial time series and improve data stationarity. First, differencing is applied to remove trend components from the series. A volatility-based dynamic weighting algorithm is then used to identify structural breaks, while periods of extreme volatility are treated using a dedicated filtering procedure. An adaptive adjustment module dynamically modifies the smoothing parameters according to market trading activity, strengthening noise filtering during highly oscillatory periods while preserving trend information during directional market phases. In addition, an adaptive window mechanism is introduced to update statistical parameters over time, allowing the preprocessing procedure to accommodate mean reversion and volatility clustering. The resulting data retain economically meaningful characteristics while exhibiting improved statistical properties, thereby providing higher-quality inputs for model training.

#### 2.2.3 Multi-scale market-state recognition module

##### 2.2.3.1 Multi-window LSTM design

The multi-scale market-state recognition module is built on a multi-window LSTM architecture. LSTM networks were originally designed to address long-term dependency problems in recurrent neural networks and are well suited to sequential representation learning (Hochreiter and Schmidhuber, 1997). Its main improvement lies in a hierarchical time-series analysis scheme designed to capture market dynamics across different temporal scales. The model employs parallel LSTM networks, with each network operating on a specific time horizon. The architecture includes three representative windows: a 5-day short-term window for characterizing near-term price fluctuations, a 20-day medium-term window for capturing trend-reversal signals, and a 60-day long-term window for identifying cyclical market patterns. Each LSTM unit receives the historical price sequence corresponding to its designated window and recursively encodes the temporal features of that sequence. As shown in Equation (11).


$$\begin{array}{c} h_{t}^{\left(w\right)}=\text{LSTM}\;\;(x_{t-w+1:t},h_{t-1}^{\left(w\right)}) \end{array}$$ (11)

Where $h_{t}^{\left(w\right)}$ denotes the hidden state of the LSTM unit at time *t* under window *w*, and $x_{t-w+1:t}$ represents the input sequence beginning at time *t-w + 1*. Each LSTM unit captures financial dynamics at its corresponding temporal scale, while the parallel architecture integrates information across multiple time horizons, thereby improving the representational capacity of the resulting market-state feature vector.

##### 2.2.3.2 Market-state classification mechanism

The market-state recognition mechanism uses the hierarchical temporal analysis module to construct a composite feature vector and applies a deep neural network to classify financial market conditions. The market is divided into three operating states: oscillating market, trending market, and high-volatility market, each corresponding to distinct market characteristics and investment strategies. During feature integration, an adaptive weighting scheme dynamically aggregates the outputs of multiple temporal LSTM units. The fused representation is then passed through a fully connected network and a Softmax function to generate a probability distribution over multiple market states, expressed mathematically as follows. As shown in Equation (12):


$$\begin{array}{c} s_{t}=\text{softmax}(W_{s}\cdot \text{concat}(h_{t}^{\left(5\right)},h_{t}^{\left(20\right)},h_{t}^{\left(60\right)})+b_{s}) \end{array}$$ (12)

Where $s_{t}$ denotes the state-probability vector at time *t*, and $W_{s}$ and $b_{s}$ denote the weight matrix and bias vector of the classification layer, respectively. The prediction mechanism is jointly optimized using the temporal state-probability model and the classifier parameters. The use of market-state information is motivated by the broader regime-switching literature, which recognizes that financial and economic time series may evolve under different underlying states rather than a single stable data-generating process (Hamilton, 1989). More recent reviews similarly indicate that machine-learning forecasting performance may vary substantially across market regimes (Cetrulo et al., 2024).

##### 2.2.3.3 Market-state transition modeling

This method uses Markov chain theory to characterize the dynamic evolution of market states through a state-transition probability matrix. The matrix contains the conditional probabilities of transitions among different states, with its parameters estimated and updated from historical data. The state-transition probabilities are calculated as follows. As shown in Equation (13):


$$\begin{array}{c} P(s_{t+1}=j|s_{t}=i)=\frac{\text{count}(\mathrm{ij})}{\text{count}(i)} \end{array}$$ (13)

Where count(ij) denotes the number of observed transitions from market state *i* to state *j*, and count(i) denotes the total number of occurrences of state *i* in the historical sequence. By statistically summarizing historical state transitions, the transition probabilities among different market states can be estimated, thereby improving the model’s ability to characterize the evolution of market conditions.

## 3 Results

### 3.1 Overall performance comparison between AHAT-Fin and iTransformer

We first use iTransformer as the primary benchmark to evaluate the overall forecasting performance of AHAT-Fin. The experimental results show that AHAT-Fin achieves improvements in forecasting accuracy, directional prediction, and risk-adjusted performance. Compared with iTransformer, AHAT-Fin reduces the mean squared error (MSE) by 20.2% on average, improves directional accuracy (DIR) by 8.4%, and increases the Sharpe ratio by 63.4%. These results indicate that augmenting the variable-wise modeling capability of iTransformer with dual-path adaptive hybrid attention, volatility-aware normalization, and market-state recognition improves both statistical forecasting performance and investment decision outcomes.

In terms of forecasting error, AHAT-Fin maintains lower MSE and MAE values across different test datasets, indicating stronger fitting and predictive performance for financial time-series dynamics. In terms of directional prediction, AHAT-Fin more accurately identifies upward and downward market movements, suggesting that the model not only reduces the numerical deviation between predicted and observed values but also captures the direction of market changes more effectively. From an investment perspective, the higher Sharpe ratio indicates that the forecasts can contribute to improved risk-adjusted investment performance. Overall, the original experimental results show that AHAT-Fin improves directional accuracy by 8.4% and the Sharpe ratio by 63.4% relative to the benchmark model.

To further assess the applicability of the model across different asset types, the original study compares representative stocks with different volatility profiles and industry characteristics. Table 1 reports the performance improvements of AHAT-Fin relative to the benchmark model across these stocks.

**Table 1:** Model performance comparison across different stocks.

Stock	MSE improvement (%)	DIR improvement (%)	Sharpe ratio improvement (%)	Market characteristics
AMD	22.5	9.2	68.7	High-volatility technology stock
JPM	18.9	7.8	61.2	Financial cyclical stock
WMT	19.4	8.1	59.8	Consumer defensive stock
CSI300001	21.1	8.9	65.4	Large-cap blue-chip stock
CSI300002	20.8	8.6	64.1	Growth stock
CSI300003	18.7	7.5	58.9	Value stock
CSI300004	19.6	8.3	62.7	Cyclical stock

As shown in Table 1, AHAT-Fin achieves varying degrees of performance improvement across assets with different market characteristics. For AMD, a high-volatility technology stock, the model reduces MSE by 22.5%, improves directional accuracy by 9.2%, and increases the Sharpe ratio by 68.7%, representing one of the largest improvements among the tested assets. Relatively consistent gains are also observed for JPM, WMT, and the different types of CSI 300 constituent stocks. These results suggest that the predictive advantage of AHAT-Fin is not confined to a single category of equities and that the model exhibits a certain degree of adaptability across industries and market characteristics.

### 3.2 Comparison with multiple benchmark models

To further evaluate the forecasting performance of AHAT-Fin, we compare it with several representative time-series forecasting models, including LSTM, GRU, Transformer, Autoformer, Informer, and iTransformer. All models use the same data preprocessing procedure, historical input window, and forecasting horizon to reduce the influence of differences in experimental settings. The original study conducts multi-model comparisons across seven financial datasets, using MSE and MAE as the primary evaluation metrics. Table 2 reports the MSE results of all models across the seven datasets, while Table 3 presents the corresponding MAE results.

**Table 2:** Multi-model comparison results (MSE).

Model	DD	HPQ	CVX	WMT	AMD	JPM	SAP
LSTM	0.0156	0.0189	0.0167	0.0145	0.0234	0.0178	0.0162
GRU	0.0152	0.0185	0.0163	0.0142	0.0229	0.0174	0.0158
Transformer	0.0148	0.0179	0.0158	0.0138	0.0221	0.0168	0.0154
Autoformer	0.0142	0.0173	0.0152	0.0134	0.0215	0.0162	0.0149
Informer	0.0139	0.0168	0.0149	0.0131	0.0208	0.0145	0.0146
iTransformer	0.0135	0.0164	0.0145	0.0128	0.0201	0.0141	0.0142
AHAT-Fin	0.0129	0.0157	0.0138	0.0122	0.0193	0.0112	0.0135

**Table 3:** Multi-model comparison results (MAE).

Model	DD	HPQ	CVX	WMT	AMD	JPM	SAP
LSTM	0.0287	0.0342	0.0298	0.0265	0.0389	0.0324	0.0276
GRU	0.0281	0.0336	0.0292	0.0259	0.0382	0.0318	0.0271
Transformer	0.0274	0.0329	0.0285	0.0252	0.0374	0.0311	0.0265
Autoformer	0.0267	0.0321	0.0278	0.0245	0.0365	0.0303	0.0258
Informer	0.0259	0.0314	0.0271	0.0238	0.0356	0.0296	0.0251
iTransformer	0.0252	0.0307	0.0264	0.0231	0.0230	0.0289	0.0245
AHAT-Fin	0.0241	0.0294	0.0253	0.0221	0.0183	0.0275	0.0234

As shown in Table 2, AHAT-Fin achieves lower MSE values than all comparison models across the seven datasets. Overall, iTransformer outperforms LSTM, GRU, and the other Transformer variants, while AHAT-Fin further reduces forecasting errors relative to iTransformer. These results suggest that incorporating temporal dependencies, dynamic volatility, and market-state information in addition to variable-wise attention can improve the forecasting performance of financial time series. All MSE values reported above are taken from the multi-model comparison experiments in the original study.

The MAE results are broadly consistent with the MSE results. AHAT-Fin achieves the lowest MAE across all seven datasets, indicating that its performance advantage is not driven by a small number of extreme forecasting errors but is also reflected in stable average absolute prediction errors. In particular, the model maintains relatively low forecasting errors for stocks such as AMD, JPM, WMT, and CVX, which differ in volatility profiles and industry characteristics, suggesting a certain degree of cross-industry adaptability.

Across model categories, LSTM and GRU exhibit relatively higher forecasting errors, while Transformer-based models generally outperform conventional recurrent neural networks. Autoformer, Informer, and iTransformer further improve long-sequence forecasting performance. Building on these advances, AHAT-Fin achieves lower MSE and MAE values, suggesting that structural adaptations targeting non-stationarity, volatility, and changes in market states can further improve financial time-series forecasting performance.

### 3.3 Ablation study result

To evaluate the individual contributions of the dual-path adaptive hybrid attention mechanism, volatility-aware normalization, and market-state recognition module, we conduct ablation experiments by removing each component separately while keeping the remaining model settings unchanged and comparing the resulting variants with the full AHAT-Fin model. The original experiments use seven financial assets-AMD, JPM, WMT, AAPL, MSFT, Kweichow Moutai, and CATL-and evaluate the performance of different model configurations using MSE, directional accuracy (DIR), and the Sharpe ratio. The corresponding results are reported in Tables 4–6.

**Table 4:** MSE under different module configurations.

Module configurations	AMD	JPM	WMT	AAPL	MSFT	Constituent Stock A	Constituent Stock B	Average change
AHAT-Fin	0.0245	0.0312	0.0198	0.0287	0.0256	0.0341	0.0298	-
w/o DPA	0.0276	0.0352	0.0223	0.0324	0.0289	0.0384	0.0336	+12.7%
w/o Vol-Norm	0.0290	0.0369	0.0234	0.0340	0.0303	0.0403	0.0352	+18.3%
w/o Market-State	0.0267	0.0334	0.0215	0.0308	0.0275	0.0365	0.0319	+7.4%

**Table 5:** Directional accuracy under different module configurations.

Module configurations	AMD	JPM	WMT	AAPL	MSFT	Constituent Stock A	Constituent Stock B	Average change
AHAT-Fin	0.642	0.618	0.675	0.658	0.634	0.601	0.625	-
w/o DPA	0.609	0.587	0.641	0.625	0.602	0.570	0.593	−5.1%
w/o Vol-Norm	0.598	0.576	0.628	0.612	0.589	0.559	0.581	−6.9%
w/o Market-State	0.598	0.576	0.629	0.614	0.591	0.560	0.583	−6.8%

**Table 6:** Sharpe ratio under different module configurations.

Module configurations	AMD	JPM	WMT	AAPL	MSFT	Constituent Stock A	Constituent Stock B	Average change
AHAT-Fin	1.23	0.98	1.45	1.18	1.12	0.87	1.03	-
w/o DPA	1.09	0.84	1.28	1.02	0.97	0.74	0.89	−18.5%
w/o Vol-Norm	1.05	0.79	1.21	0.96	0.91	0.69	0.83	−23.7%
w/o Market-state	0.71	0.56	0.83	0.68	0.64	0.50	0.59	−42.6%

Removing any of the three core modules leads to an increase in MSE. In particular, removing the volatility-aware normalization module results in the largest increase in average MSE, at 18.3%. Removing the dual-path attention mechanism increases average MSE by 12.7%, while removing the market-state module increases it by 7.4%. These results indicate that all three modules contribute to forecasting accuracy, with the volatility-aware normalization module playing a particularly important role in reducing numerical prediction errors.

In terms of directional prediction, the full model achieves the highest DIR across all seven financial assets. Removing the dual-path attention mechanism reduces directional accuracy by an average of 5.1%, while removing the volatility-aware normalization module and the market-state recognition module leads to average decreases of 6.9 and 6.8%, respectively. These results indicate that all three modules contribute to the model’s ability to predict market direction.

Compared with its effect on forecasting errors, the market-state recognition module has a more pronounced impact on risk-adjusted performance. Removing this module reduces the Sharpe ratio by an average of 42.6%, while removing the volatility-aware normalization module and the dual-path attention mechanism leads to average decreases of 23.7% and 18.5%, respectively. These results indicate that the three core modules play different functional roles within the full model: volatility-aware normalization contributes more strongly to reducing forecasting errors, whereas market-state recognition has a comparatively greater influence on risk-adjusted investment performance.

### 3.4 Visualization of attention weights

To further examine the information allocation patterns of the dual-path adaptive hybrid attention mechanism under different market conditions, we visualize the attention weights along both the variable and temporal dimensions. The corresponding attention heatmaps are shown in Figure 1.

**Figure 1:**
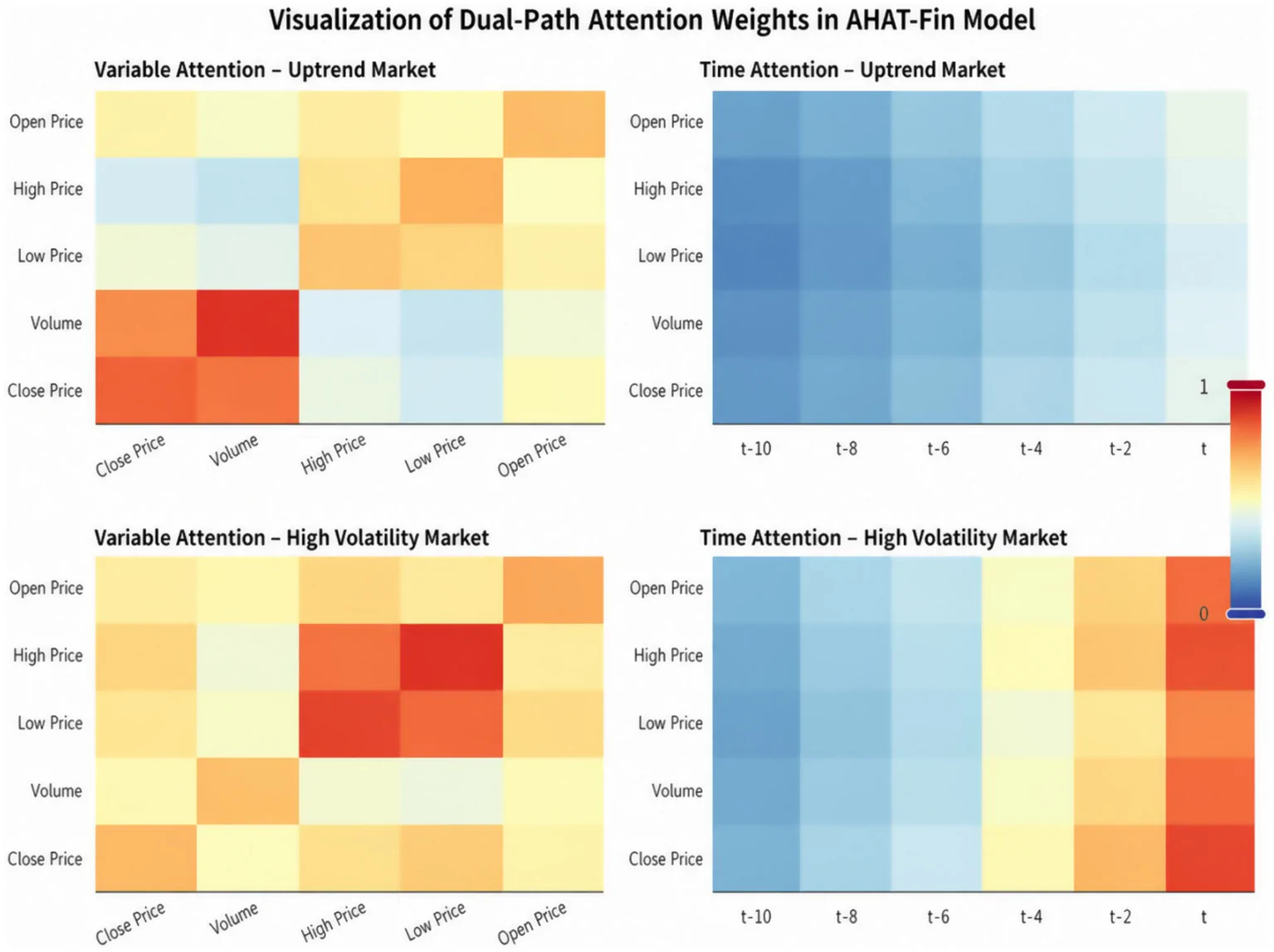
Visualization of attention weights.

The heat maps show that the combinations of financial variables emphasized by the model vary across market environments. During upward market phases, relatively high attention weights are assigned to trading volume and closing price, indicating that the model places greater emphasis on volume–price interactions in trending markets. When the market enters a highly volatile phase, greater attention is assigned to the intraday price range between the high and low prices, suggesting that changes in market conditions lead to dynamic adjustments in variable importance.

The distribution of attention weights along the temporal dimension also exhibits clear state-dependent differences. During sustained upward movements, attention is concentrated more heavily on the current time step and adjacent recent observations, indicating that the model relies more on recent market information to extend trend judgments. In contrast, during periods of intense market fluctuations, the attention weights become more dispersed, and some earlier historical observations receive higher weights. This suggests that AHAT-Fin adjusts its use of recent and longer-horizon historical information across different market states.

Overall, the attention weights do not remain fixed throughout the sample period; instead, they vary with changes in market conditions. This finding indicates that the dual-path adaptive attention mechanism performs dynamic information selection along both the variable and temporal dimensions.

### 3.5 Market-state recognition results

The multi-scale market-state recognition module extracts market features using 5-day, 20-day, and 60-day windows and classifies market conditions into three categories: range-bound, trending, and high-volatility states. The experimental results show that the module achieves relatively strong recognition performance across all three market states. The detailed market-state recognition results are reported in Table 7.

**Table 7:** Market-state recognition accuracy.

Market state	Recognition accuracy (%)	Misclassified as range-bound (%)	Misclassified as trending (%)	Misclassified as high-volatility (%)
Oscillating market	87.2	–	8.4	4.4
Trending market	90.5	6.8	–	2.7
High-volatility market	84.8	7.2	8.0	–

The empirical results further demonstrate the effectiveness of the system under different market conditions. During range-bound periods, the model captures price movements within the support and resistance range, achieving a classification accuracy of 87.2%. In trending markets, the multi-window analysis framework attains recognition accuracies of 89.6% for upward trends and 91.3% for downward trends. Under extreme volatility, the system also maintains relatively strong performance, correctly identifying high-volatility regions with an accuracy of 84.8%.

Taken together, the experimental results show that AHAT-Fin exhibits consistent advantages across benchmark comparisons, cross-asset tests, ablation studies, and market-state recognition experiments. The multi-model comparison indicates that AHAT-Fin generally achieves lower MSE and MAE values than the benchmark models. The ablation results further show that the dual-path attention mechanism, volatility-aware normalization, and market-state recognition module contribute to forecasting accuracy, directional prediction, and risk-adjusted performance, respectively. In addition, the attention-weight visualizations and market-state recognition results provide further empirical evidence of the model’s ability to adapt dynamically to different financial market conditions.

## 4 Discussion

The results of this study suggest that jointly accounting for cross-variable dependencies, temporal dynamics, time-varying volatility, and market-state changes can improve financial time-series forecasting under heterogeneous market conditions. Compared with the baseline iTransformer, AHAT-Fin reduces the mean squared error by 20.2% on average, improves directional accuracy by 8.4%, and increases the Sharpe ratio by 63.4%. Improvements are also observed across assets with different industry characteristics and volatility profiles. Rather than indicating that one architectural modification alone drives the performance gains, the ablation results show that the three components of AHAT-Fin contribute in different but complementary ways. This distinction is important because forecasting accuracy, directional prediction, and risk-adjusted performance do not respond equally to each component.

The dual-path adaptive hybrid attention mechanism mainly improves the representation of multidimensional financial information. Conventional Transformer-based forecasting models typically emphasize either temporal dependencies or relationships across variables. In financial markets, however, the relevance of these two information dimensions may change with market conditions. The attention-weight visualizations in this study show that the model does not maintain a fixed allocation of attention throughout the sample. During trending periods, greater weight is assigned to recent observations and volume–price relationships, whereas during highly volatile periods, attention becomes more dispersed and the model places greater emphasis on intraday price ranges and earlier historical observations. The ablation results support this interpretation: removing the dual-path attention mechanism increases average MSE by 12.7% and reduces directional accuracy by 5.1%. These findings are consistent with recent evidence that dual-attention architectures can improve financial time-series forecasting by jointly modeling heterogeneous variables and temporal structures (Hadizadeh et al., 2025). The present results further suggest that the benefit of such architectures may lie not only in extracting information from two dimensions, but also in allowing their relative importance to vary with market conditions.

A different pattern emerges for volatility-aware normalization. Removing this module produces the largest deterioration in numerical forecasting accuracy, increasing average MSE by 18.3%. This result is consistent with a basic characteristic of financial data: the scale and variance of returns are not constant over time but exhibit conditional heteroskedasticity and volatility clustering. Fixed normalization parameters may therefore become less appropriate when the market moves between relatively stable and highly volatile periods. Previous studies have shown that combining econometric volatility structures with neural networks can improve volatility and risk forecasting (Buczynski and Chlebus, 2024; Petrozziello et al., 2022). AHAT-Fin differs from these approaches because GARCH-related volatility information is not used as a separate forecasting output. Instead, it is incorporated into the feature-processing stage to adjust the representation of the input data. The ablation evidence indicates that introducing financial volatility information before or during representation learning can be useful for reducing forecasting errors in non-stationary financial series.

The market-state recognition module appears to play a different role. The module identifies range-bound, trending, and high-volatility states using 5-day, 20-day, and 60-day windows, with recognition accuracy exceeding 80% for all three market conditions and reaching 90.5% in trending markets. More importantly, removing this module reduces the Sharpe ratio by an average of 42.6%, substantially more than removing either the dual-path attention mechanism or volatility-aware normalization. This result suggests that state information is especially relevant when forecasts are translated into investment decisions. Market relationships learned under one regime may not remain equally informative after a change in volatility, trend direction, or dependence structure. Research on financial prediction under regime change has similarly emphasized that machine-learning performance can vary across market environments and that adaptive mechanisms are needed when the underlying data-generating process changes (Cetrulo et al., 2024). In this sense, the contribution of market-state recognition in AHAT-Fin is not simply to create an additional classification task, but to provide contextual information that allows the forecasting framework to respond differently across market environments.

Taken together, the three components therefore appear to address different sources of difficulty in financial forecasting. Dual-path attention primarily improves information extraction across variables and time; volatility-aware normalization improves adaptation to changing data scales and volatility conditions; and market-state recognition provides information about the broader market environment in which predictions are made. Their complementary effects help explain why AHAT-Fin improves not only MSE and MAE but also directional accuracy and the Sharpe ratio. This also suggests that evaluating financial forecasting models solely on point-error measures may provide an incomplete picture. A model that achieves a small reduction in numerical error may not necessarily generate better directional signals or more stable risk-adjusted performance, particularly when market conditions change.

The results also have implications for the relationship between AI-based forecasting and financial risk management. Recent research has increasingly extended deep learning from price prediction toward volatility forecasting, VaR, Expected Shortfall, and other measures of financial risk. For example, GARCH Net combines GARCH structures with neural networks for VaR forecasting, while other neural-network approaches have been evaluated using volatility, VaR, and ES measures (Buczynski and Chlebus, 2024; Sahiner et al., 2023). Transformer-based architectures have likewise shown potential for financial volatility forecasting and risk assessment (Mishra et al., 2024). The current study contributes to this direction by incorporating volatility and market-state information into the forecasting process and by evaluating risk-adjusted investment performance through the Sharpe ratio. However, this distinction should be maintained carefully. AHAT-Fin is primarily a financial time-series forecasting framework with volatility- and market-state-aware mechanisms; it is not, in its current form, a complete VaR- or ES-based risk-management model. The observed improvement in the Sharpe ratio therefore provides evidence of potential decision value rather than direct evidence of superior tail-risk measurement.

Several limitations qualify these findings. First, the additional modules increase model complexity, and the original experiments indicate an approximately 35% increase in training time. This may limit deployment in applications requiring frequent updates or low-latency inference. Second, although the model performs well under the three predefined market states, its recognition accuracy for black-swan events is approximately 62%, indicating that rare events outside the historical distribution remain difficult to identify. Third, the empirical analysis is mainly based on structured equity-market data from the United States and China. Although the cross-asset results indicate some degree of adaptability, they do not establish that the model will retain the same performance in foreign exchange, commodity, crypto-asset, or other market settings. Finally, historical-window-based normalization and state recognition may respond slowly to persistent structural changes, while the current input variables do not explicitly incorporate macroeconomic conditions, policy information, news, or market sentiment.

These limitations suggest several closely related extensions rather than separate research directions. Future work could reduce computational costs through lightweight attention structures, model compression, or parameter sharing, while online learning and dynamic parameter updating could improve adaptation to structural breaks and concept drift. Market-state recognition could also be extended beyond the current three-state framework by incorporating anomaly detection, macroeconomic variables, policy signals, and sentiment information. Broader tests across asset classes would help establish the external validity of the framework. Most importantly for financial risk applications, future studies could incorporate VaR, Expected Shortfall, and formal backtesting procedures to determine whether the benefits observed in forecasting accuracy and the Sharpe ratio also extend to tail-risk prediction and risk-control performance.

## 5 Conclusion

This study proposed AHAT-Fin, an adaptive hybrid attention and market-state-aware framework for financial time-series forecasting. By integrating dual-path attention, GARCH-based volatility adjustment, and multi-window LSTM market-state perception, the framework jointly captures temporal dependencies, inter-variable relationships, time-varying volatility, and transitions among oscillating, trending, and abnormal high-volatility states.

Experiments on daily data from U. S. stocks and CSI 300 constituent stocks show that AHAT-Fin generally outperforms the benchmark models. On average, it reduces mean squared error by 20.2%, improves directional accuracy by 8.4%, and increases the Sharpe ratio by 63.4%. Ablation results confirm that all three components contribute to the overall performance, with market-state perception making a particularly important contribution to risk-adjusted investment performance. Attention and market-state visualizations further support the model’s interpretability.

The current study is mainly based on daily stock data; therefore, the applicability of AHAT-Fin to other asset classes and high-frequency data requires further validation. Such extensions may require adaptations for market microstructure noise, transaction costs, irregular trading activity, and larger data volumes. Future research will also examine the sensitivity of multi-window settings and incorporate additional information, such as investor sentiment and macroeconomic indicators, to improve the framework’s generalizability and practical scalability.

## Data Availability

The datasets presented in this study can be found in online repositories. The names of the repository/repositories and accession number(s) can be found in the article/supplementary material.
